# The Role of Diet in Regulation of Macrophages Functioning

**DOI:** 10.3390/biomedicines10092087

**Published:** 2022-08-26

**Authors:** Yurgita R. Varaeva, Tatiana V. Kirichenko, Nataliya N. Shaposhnikova, Dmitry B. Nikityuk, Antonina V. Starodubova

**Affiliations:** 1 Federal Research Centre of Nutrition, Biotechnology and Food Safety, 109240 Moscow, Russia; 2 Petrovsky National Research Centre of Surgery, Avtsyn Research Institute of Human Morphology, 119991 Moscow, Russia; 3 Chazov National Medical Research Center of Cardiology, 121552 Moscow, Russia; 4 Therapy Faculty, Pirogov Russian National Research Medical University, 117997 Moscow, Russia

**Keywords:** diet, biologically active compounds, macrophages

## Abstract

The great importance of diet for health and high life-expectancy is established. The impact of nutrients on immune system is a point of growing research interest. Recent studies have found pro- and anti-inflammatory properties of some diet patterns and nutrients that can be used from the bench to the bedside for chronic low-grade inflammatory status correction. In this regard, the assessment of potential effects of nutrition on macrophage differentiation, proliferation, and functioning in health and disease is highly demanded. In this review, we present current data on the effects of nutrients on the macrophage functioning.

## 1. Introduction

The question about the ability of diet to change macrophage functioning was first was raised in the 1970s. Early studies, from the 1970s to 1990s, were devoted to the investigation of the effects of different diet patterns and vitamin deficiencies, including cholesterol-rich diet [1,2], protein-deficient diet [3], and vitamin B6-deficient diet [4]. In the early period, the functioning of intestinal macrophages raised special interest [5] because of their crucial role in intestinal homeostasis. Currently, the point of interest has been shifted to the role of intestinal macrophages as a key factor in inflammation [6,7,8]. In the past 20 years, the number of studies has risen dramatically, and their focus has expanded from diet patterns to food products, nutrients and compounds, the effects on macrophage recruitment, functions, and M1 or M2 phenotype activation [9,10,11]. Macrophages play a key role in inflammation, regulation of immune response, and tissue repair. Based on their origin, macrophages are divided into blood monocyte-derived ones and resident tissue macrophages. There are several different subsets of macrophages based on their anatomical location, in particular, intestinal macrophages in gut, osteoclasts in bones, alveolar macrophages in lung, histiocytes in the interstitial connective tissue, microglia in brain, etc. [12]. When stimulated, macrophages adopt an appropriate phenotype to carry out host antimicrobial defense, antitumor immunity and inflammatory responses. Classically activated macrophages (M1) are phagocytes that provide the host defense from a variety of pathogens, while alternatively activated macrophages (M2) have anti-inflammatory functions and regulate wound healing [13]. The phenotype of macrophages switches under the influence of various local microenvironment signals [14]. Numerous studies demonstrate the effects of nutrients and metabolites on macrophages in immune and inflammatory pathways [15]. In this review, we present current data on the effects of nutrients on macrophage functioning.

## 2. The Role of Diet Patterns and Food Groups

The diet patterns’ effect on physiological and pathological processes is of particular interest because of intervention results being close to “real life”. Otherwise, the assessment of the whole diet is highly demanded in study design, and it requires a complicated method, precise power calculations with massive sample sizes, and long follow-up periods. Table 1 demonstrates the results of recent studies which investigated the association of diet patterns with macrophage functioning.

Western diet. A high-fat, high-sugar, high-salt Western diet is associated with a negative health impact including cardio-vascular diseases, obesity, diabetes, oncology, etc. The pro-inflammatory pathological pathway is one of the key negative factors. Pro-inflammatory activation of macrophages induces their infiltration into adipose tissue as a part of low-grade inflammation and insulin resistance development. So, it was shown in a model of male C57BL/6 J mice fed on a 60% high-fat diet (HFD) vs. a 10% fat normal diet that an HFD induced acute reduction in blood monocyte count at the early stages of HFD with subsequent dramatic fat expansion and accumulation of adipose tissue macrophages [16]. High fat consumption causes increased caspase-1 activation and IL-18 expression in macrophages that promote the insulin resistance and, subsequently, increase the obesity risk. Obesity is a well-known inducer of low-grade inflammation widely used to cause steatohepatitis and type 2 diabetes in animal models [26,27].

Numerous studies demonstrate the pro-inflammatory stimulation effect of HSD on different types of macrophages [28]. It was shown that high salt content enhances the pro-inflammatory properties of a Western diet by activation of proliferation of classical pro-inflammation phenotype (M1) macrophages and suppression of macrophage autophagocytic capacity [18]. In a murine model of experimental autoimmune encephalomyelitis, an HSD induced pro-inflammatory differentiation of macrophages due to increased activation of nuclear factor kappa-B (NF-kB), and mitogen-activated protein kinase (MAPK) signaling pathways resulted in significant disease aggravation [29]. Another study in mouse models of acute lung injury shows that HSD increased proinflammatory gene expression of mouse alveolar macrophages as well as potentiated LPS-induced pro-inflammatory activation of macrophage and suppressed IL-4-induced anti-inflammatory activation of macrophages [19]. Moreover, in mice on HSD, hypertonic conditions lead to activation of inflammasomes via mitochondrial reactive oxygen species (ROS), and induced IL-1β secretion through caspase-1 activation in macrophages [30]. Despite the fact that high salt intake is considered as one of the major risk factors that enhances morbidity and mortality in developed countries, some studies report the beneficial effects of the sodium pro-inflammatory potential including its effect on macrophages. So, HSD can stimulate the immune response, especially in terms of antimicrobial effect. In particular, enhanced sodium content in the skin by HSD boosted activation of macrophages in an Nfat5-dependent manner promoting cutaneous antimicrobial defense [31]. In another study, HSD promoted production of interferon via the p38 MAPK/ATF2/AP1 signaling pathway increasing antiviral resistance [32]. However, the immunostimulatory or immunosuppressive effects of salt currently require further study.

The high intake of red and processed meat is another feature of Western-style diet that causes rise in low-grade inflammation rates [33] according to UK-Biobank data, unless evidence of associated pro-inflammation macrophage activation is limited.

Protein restriction introduces the ability to reduce the macrophage proliferation. The ability of protein or/and amino acids diet restriction to reduce the proliferation of pro-tumor M2 macrophages was investigated in macrophages cell-lines from murine tumor-models [20]. Due to the pathological mechanisms mentioned above, the Western diet is considered as a pro-inflammatory diet pattern with exceptional negative health effects.

The Mediterranean-type diet (MTD) is a diet pattern that, in a great number of studies, shows the ability to increase life expectancy, and to reduce cardiovascular disease (CVD), type 2 diabetes, obesity, oncology, and obesity-related systemic inflammation [34] because of a wide range of pathways [35], including reduction in macrophage pro-inflammatory cytokines secretion [22]. MTD also is also distinguished by the unique combination of oleic acid and anthocyanins-rich products that, in in vitro studies, perform the reduction in pro-inflammatory (e.g., MCP-1, TNF-α, IL-6, and IL-1β mRNA) macrophage status and increase the production of anti-inflammatory markers [35].

The described effects of the MTD are based on a unique combination of high extra virgin olive oil (EVOO), oily-fish and vegetables consumption. EVOO has in vitro proven anti-inflammation effects of polyphenols on macrophages [36,37], including the reduction in NO release and iNOS expression. Fish oil in human studies represents the inhibition of inflammation-related genes expression in macrophages [38] and fresh vegetables, especially cruciferous, are pointed out by outstanding anti-inflammatory properties through biological-active compounds, which promote the M2 (anti-inflammatory) type of macrophage proliferation [39]. Nowadays, the MTD is one of the diet patterns with evidence-based positive effects on population health and life-expectancy. Anti-inflammatory properties of MTD also provide cognitive protection [40,41], which was used in the development of the Mediterranean-DASH Intervention for Neurodegenerative Delay (MIND) diet with neuroprotective effect [40,42]. The effects of the MIND diet are explained by a wide range of natural antioxidants taking part in neuromembrane stabilization and compensation of active microglia inflammation and ROS synthesis [43]. Nowadays, some study describe the anti-inflammation neuroprotective effects of polyunsaturated fatty acids (PUFAs) through NF-κB and the mitogen-activated protein kinases (MAPK) pathway in microglia [44]. Another featured nutrient of the MIND diet, hydroxytyrosol (EVOO derivate) decrease LPS-induced microglia activation [45]. Thus, the MIND diet plays an anti-inflammation role for the microglia through the same pathways as MTD on macrophages as a whole.

The Nordic diet includes increased content of berries, fish, nuts, and seeds with limited consumption of red and processed meat, sugar, salt and alcohol [46]. This diet was shown to decrease the low-grade inflammation [47], suppress the platelet activation [48], and reduce the risk of CVD and stroke [49]. Berries are the key feature of the Nordic diet. According to recent studies, regular berries intake reduces systemic inflammation [50] and oxidative stress factors. In human research, berries and berries extracts show the reduction in TNF-α levels [51], inhibition of NF-kB and MAPK cascades, nitric oxide (NO) synthesis reduction [52]. The outstanding interest relates to blueberries. A reduction in TNF-α and CD-11 genes expression was described in murine mesenteric fat on blueberry enriched diet [50]. Overall, the anti-inflammatory protective effect of blueberries was established in a wide range of research fields, from atherosclerosis [53] to tumor growth [54].

More studies are needed to investigate the effects of another reliably healthy diet-pattern—Dietary Approach to Stop Hypertension (DASH diet). In a cross-sectional study performed on 305 obese and overweight women, the DASH diet was not shown to affect the levels of several inflammatory factors including monocyte chemoattractant protein-1 (MCP-1), the key chemokine regulating the migration and infiltration of macrophages [25]. However, there are currently too few studies evaluating the effect of the DASH diet on macrophage function to draw any conclusions.

The influence of different diet patterns and nutrients is especially relevant in the context of intestinal macrophages, since dietary factors are able to modify the profile of intestinal macrophages, affecting their key functions in the gut homeostasis [55]. HFD was shown to activate the MCP-1/CCR2 axis in intestinal macrophages in subjects with colorectal cancer, while the higher expression of CD163, the marker of M2 macrophages, was demonstrated on the same cohort [17]. Higher expression of inflammatory mediators such as transforming growth factor-β (TGF-β), TNF-α and IL-1β in intestinal macrophages was detected in the animal protein group in the murine model of immuno-competent BALB/c and immuno-deficient RAG2 knock-out mice on high animal protein diet vs. high plant protein diet [21]. A lot of studies described in the next section are devoted to the estimation of the impact of various nutrients on the function of intestinal macrophages. It was shown that short-chain fatty acids (SCFAs) exert immunomodulatory effects on intestinal macrophages. The treatment of macrophages with butyrate, which is one of the key metabolites of gut microbiota, in particular, phyla Bacteroidetes and Firmicutes, leads to the suppression of LPS-induced pro-inflammatory mediators, such as NO, IL-6, and IL-12 [56]. Intestinal macrophages occupy an important place in the diet–microbiota axis; however, it demands a separate review to cover this topic.

## 3. The Role of Micronutrients and Biologically Active Compounds

A major part of current studies is devoted to the assessment of micronutrients and biologically active compounds’ influence on macrophage proliferation and functioning. These studies use a reliable and reproducible design of inclusion or exclusion compounds for diet or cell treatment with an assessment of markers’ (inc. gene expression, biomarkers levels, enzyme activity, cell morphology, histology, etc.) reaction to intervention. As intervention usually involves a single pure compound, the results are reliable and precisely describe the effect of the compound (Table 2).

### 3.1. Functional Amino Acids

Dietary amino acids might be involved in the regulation of homeostasis of intestinal macrophages. Several studies in murine model demonstrate that methionine- and choline-deficient diet (MCD) possesses a significant pro-inflammatory potential. So, mRNA levels of pro-inflammatory mediators IL-1B, IL-6, TNF-, and MCP7-1 were significantly higher and intestinal mRNA levels of anti-inflammatory cytokines IL-4 and IL-13 were significantly lower in MCD-fed mice [57]. Another study suggests that functional amino acids are one of the regulatory factors for intestinal macrophage functioning, including the potential to decrease IL-10 synthesis [58].

**Table 2:** Recent studies investigated the association of micronutrients and biologically active compounds with macrophage functioning.

Intervention	Year	Authors	Population	Brief Results (Effects of the Intervention)
Functional amino acids	2016	Ochi T. et al. [58]	In vivo—Mice.	Selective deprivation of IL-10 production in intestinal macrophages.
Dietary fiber	2020	Jang Y.O. et al. [59]	In vivo—Eight-week-old inbred female C57BL/6 mice emphysema model on 20% dietary fiber diet and SCFAs supplementation.	Significant reduction in the number of macrophages in the broncho-lavage fluids and the serum IL-1β, TNF-α, IL-6, IL-8, IL-18, IRF-5, TGF-β, and MMP-12 mRNA levels on high-fiber diet that is usually associated with macrophages infiltration. Reduction in IL-6 and IFN-γ levels in the broncho-lavage fluids on SCFAs supplementation.
Inulin	2020	Wang Z. et al. [60]	In vivo—60 female C57BL/6J mice, incl. alcohol-fed group. In vitro—murine RAW264.7 macrophages cell-line.	In vivo: Reduction in iNOS and TNF-α levels (M1 activity). Increase in IL-10b and Arg-1 (M2 activity) in Inulin supplementation in alcohol-fed mice. In vitro: Described effects were associated with SCFAs content in inulin.
SCFA n-butyrate	2014	Chang, P.V. et al. [56]	In vitro—Intestine macrophages cell-line.	Modulation of intestine macrophages functioning through the downregulation of LPS-inducedNO, IL-6, and IL-12 production via the histone deacetylase inhibition.
	2018	Trompette A. et al. [61]	In vivo—Female C57BL/6 mice infected with influenza A on SCFAs treatment.	Induction of Ly6c+ patrolling monocytes proliferation in lungs to alternate macrophages CD206 profile and increase IL-4 and IL-10 mRNA levels.
Butyrate and propionate	2021	Huang C. et al. [62]	In vivo—Female C57BL/6 mice. In vitro—Murine alveolar MH-S cell-line.	In vivo: Decrease in M2 phenotype proliferation. In vitro: Dose dependent inhibition of M2-associated gene expression.
PUFA	2012	Liu Y. et al. [63]	In vivo—24 weaned pigs, 21 days on diet.	Decrease in TNF-α expression.
	2020	Hutchison A. et al. [64]	In vitro—Splenic CD11b+ macrophages co-cultured with L6 myocytes from male Sprague Dawley rats’ cell-line treated with HFD with ω-3 or ω-6 PUFAs for 2, 8 or 12 weeks.	Reduction in LPS-induced mRNA expression of TNF-α in a cell-line treated with ω-3 PUFAs.
Alpha-linolenic acid (ALA), eicosapentaenoic acid (EPA), and docosahexaenoic acid (DHA)	2020	Li Q. et al. [65]	In vitro—Macrophages from large yellow croaker LPS-activated cell-line.	Inhibition of TLR2, TLR5 and PGLYRT5 genes. Decrease in LPS-induced phosphorylation of IKKα/β, especially for DHA. DHA inhibits the synthesis of phosphorylated p38 induced by LPS.
Arachidonic acid (AA), eicosapentaenoic acid (EPA), and docosahexaenoic acid (DHA)	2020	Fournier N. et al. [66]	In vitro—J744A.1 murine cholesterol-loaded macrophages cell-line a cholesterol loaded human macrophages cell-line (HMDM).	Dose-dependent reduction in cholesterol efflux via the cardioprotective ABCA1 pathway in murine AA and EPA (in less extend) groups. Dose-dependent reduction in cholesterol efflux via the cardioprotective ABCA1 pathway in human EPA, DHA, and AA (in less extend) groups.
Oleic Acid	2021	Santamarina A.B. et al. [35]	In vitro—THP-1-derived monocytes and macrophages cell-line	Decrease in the pNFκBp65, PPARγ, IκBα, TNF-α, IL-1β, IL-6, and MCP-1. Increase in IL-10 production.
Vitamin D	2011	Fabri M. et al. [67]	In vitro—Cell-line.	Activation of IFN-γ-induced *M. tuberculosis*-infected macrophages.
	2012	Zhang Y. et al. [68]	In vitro—Monocytes cell-line.	Suppression of IL-6 and TNF-α through MAPK phosphatase-1 inhibition.
	2018	Gunasekar P. et al. [69]	In vivo—9 Yucatan female microswine of 30–40 lbs (obese atherosclerosis model) on vitamin D deficient (500 IU daily), sufficient (2500–3500 IU daily) or supplemented (4500–5500 IU daily) diet for 12 months.	Increase in CD206 M2 macrophages expression in epicardial adipose tissue on Vitamin D supplemented diet.
	2018	Giraldo D.M. et al. [70]	In vivo—20 healthy volunteers randomized to 1000 or 4000 IU of vitamin D daily subscription for 10 days. In vitro—peripheral blood mononuclear cells from participants (before and after treatment).	Higher resistance of monocytes to Dengue virus on 4000 IU vitamin D daily intake. Lower dengue virus stimulated levels of hIL-6, hIL-8, hTNF-α on 4000 IU vitamin D daily intake. Higher dengue virus stimulated levels of hIL-10 on 4000 IU vitamin D daily intake. Decrease in TLR and CAMP mRNA expression on 4000 IU vitamin D daily intake.
	2019	Arboleda J.F. et al. [71]	In vitro—human monocyte-derived macrophages cell-line infected by Dengue virus treated with or without vitamin D.	Higher resistance dengue virus caused by 11 miRNA associated with vitamin D treatment. Predominant role of miR-155-5p decreases that lower SOCS-1 expression (key part of TLR4 signaling) in combination with down-regulation of TLR4, which consecutively lower NF-kB stimulated IL-1β secretion.
(Calcitriol)	2020	Niu L. et al. [72]	In vitro—U937-derived macrophages cell-line infected with *Porphyromonas gingivalis* treated with Calcitriol.	Increase in *P. gingivalis* localization in phagosomes and lysosomes, degradation of live bacteria.
(1,25(OH)D3)	2021	Small A.G. et al. [73]	In vitro—human macrophages cell-line differentiated in the presence of 1,25(OH)D3.	Increase in the complement receptor immunoglobulin (CRIg) mRNA and protein expressions (innate immunity biomarker).
Vitamin A (Retinoids)	2007	Wnag X. et al. [74]	In vitro—Cell-line.	Induction of the mRNA expression of IL-10, IL-12p40, TNF-α, IL-18, TGF-b. Reduction in IL-12 and TNF-α, but an increase in IL-10 in LPS-activated macrophages.
(β-carotene)	2022	Melnikov N. et al. [75]	In vivo—*Ldlr*−/−, *Apoe*−/−, and *db/db* mice models on HFD with or without Vitamin A supplementation.	Decrease in HFD-induced MCP-1 and CD68 mRNA in white adipose tissue. Reduction in adipose tissue macrophages recruitment.
(All-trans retinoic acids (ATRA))	2022	Babunovic G.H. et al. [76]	In vivo—Human primary monocyte-derived macrophages *Mycobacterium tuberculosis* infected, treated with ATRA	Increase in bacterial control (Mtb restriction) through decrease in intracellular cholesterol accumulation—cholesterol starvation.
Inositol hexaphosphate	2021	Wee Y et al. [77]	In vitro—Murine bone marrow macrophages cell-culture.	Increase in the development of macrophages from 0 to 2A type. Reduce LPS-induced proliferation and pro-inflammation cytokines genes expressions.
Polyphenols (Polyphenol-rich plant extract)	2019	Aires V. et al. [78]	In vivo—Wild-type C57BL/6Rj male mice, comparison of STD diet, high-fat/high-sucrose diet and high-fat/high-sucrose + Polyphenol-rich plant extract diet.	Polyphenol-enriched diet reduces the expression of pro-inflammatory macrophage genes.
Shanxi-aged vinegar extracted polyphenols (benzenepropanoic acid, benzoic acid, cinnamic acid and others, total 19 polyphenols)	2021	Du P. et al. [79]	In vitro—Cell-line RAW 264.7 LPS-activated macrophages mice model.	In vitro: Decrease in TNF-α production and nucleus and cell damage. Increase in mitochondrial membrane potential in LPS-activated cells. Dose-dependent decrease in cytokine response (IL-1β, IL-6, IL-18) in LPS-activated macrophages. Downregulation of iNOS, COX-2, p38, JNK, ERK1/2 expression. In vivo: Dose-dependent decrease in COX-2, NOS, IL-1β, IL-6, NO and TNF-α blood indexes, down-regulation of iNOS expression.
Lonicera caerulea berry polyphenols (active components—chlorogenic acid, cyanidin-3-glucoside and catechins)	2019	Liu S. et al. [80]	In vitro—Cell-line RAW 264.7 macrophages.	Dose-dependent inhibition of lipid accumulation (ox-LDL) in macrophages through the activation of Sirtuin 1 receptor and activated expression of cholesterol efflux genes ABCA1, SREBP2, miR-33.
Saskatoon berry powder (cyanidin-3-glucoside as active component)	2021	Zhao R. et al. [81]	In vivo—C57BL/6J mice obesity model High-Fat High-Sucrose diet induced.	Supplementation significantly reduces the adhesion of monocytes to aortic intima and macrophages deposition (CD-163) in the liver. Both (berry powder and pure cyaniding-3-glucoside) reduce the TNF-α, MCP-1 and PAI-1 levels but not to normal levels.
Andean berry juice (polyphenols rich: gallic acid, ellagic acid and cyaniding chloride; rich in anthocyans (Delfinidin), flavonoids (Rutin) and tannins)	2020	Arango-Varela S.S. et al. [52]	In vitro: Cell-line, LPS-stimulated RAW 264.7 macrophages (also in combination with aspirin).	Without aspirin: reduce IL-1b, MCP-1 and granulocyte-colony stimulated factor. Decrease intracellular ROS. Increase antioxidant capacity (comparable in berry juice and combined with aspirin groups). Lower NO production. Significantly increase CCL5 and IL-1α. Pure Gallic acid reduces the NO even lower than in not LPS-stimulated cell culture. Overall berry juice inhibits the CCR1 and 5 as a part of MAPK cascade that the binding to receptors caused in silico.
Flavonoids and anthocyans extract from yellow and purple corn	2018	Valenza A. et al. [82]	Hml-RFP/CyO Drosophila line.	Reduce macrophages infiltration into fat tissue. Reduce activation of JNK/SAPK p46 stress kinase—chronic inflammation marker.
Red clover extract (RC) and red clover anthocyanins fraction (RCA) (the list of 27 polyphenols (including 4 unknown compounds) and 7 anthocyans).	2020	Lee S.G. et al. [83]	In vitro—RAW 264.7 LPS-activated macrophages cell-line.	RC and RCA treatment decrease expression of IL1β, iNOS, MCP1, COX2 genes. RCA reduce the NOX1 gene induction in LPS-activated macrophages to lower levels than in non-activated macrophages and reduce NRF2 transcription factor levels to comparable to non-activated cells ones.
Anthocyans (3 compounds) and protein-bound anthocyanin compounds from sweet potato (17 protein group)	2020	Jiang T. et al. [84]	Cell-line—RAW 264.7 LPS-activated macrophages.	Dose-dependent reduction in LPS-induced NO and TNF-α production and iNOS and TNF-α mRNA expression. Increase in HO-1 mRNA and NRF2 levels (oxidative stress reduction). Significant reduction in intracellular ROS levels. Reduction in JNK, NF-kB and c-Jun protein levels—cytokine and inflammation mediators synthesis suppression.
Anthocyanins from red and purple maize	2019	Zhang Q. et al. [85]	In vitro: Cell-line—RAW 264.7 macrophages and 3T3-L1 pre-adipocytes LPS activated. Control—pure cyanidin-3-O-glucoside (C3G), peonidin-3-O-glucoside (P3G), and pelargonidin-3-O-glucoside (Pr3G). In silico: molecular docking model	Dose-dependent NO and PGE2 secretion reduction. Inhibition of the phosphorylation of JNK activated by LPS—the JNK\MAPK signaling cascade. Reduction in adipocyte TNF-α production and ROS generation. Significant decrease in the intracellular triglycerides and FAA levels. Potential decrease in the NF-kB activation—lower the pro-inflammation gene expression by inhibition of p65 nucleus accumulation (C3G and Pr3G). In silico: anthocyans show high binding affinity with 5-LOX, iNOX, COX-2 and PLA_2_ enzymes involved in NO, PGE2 and eicosanoids production.
Anthocyanin-rich aronia berry extract		Yu S-Y. et al. [86]	In vivo: male C57BL/6J mice, 14 weeks on High-Fat and High-Sucrose with or without Anthocyanins comparing to Low-Fat diet. In vitro: RAW264.7 macrophages and BMD macrophages.	In vivo and in vitro: decrease in the LPS-activated phosphorylation of p65 unit of NK-kB signaling. In vivo: significant decrease in LPS-activated mRNA expression of iNOS, COX2, TNF-α, MCP-1. In vitro: reduce the mRNA expression of Cd11b and TNF-α.
Oleic acid and anthocyanin keracyanin	2021	Santamarina A.B. et al. [35]	In vitro—Macrophages cell-line.	Keracyanin treatment decreases the pNFκBp65, PPARγ, IκBα, TNF-α, IL-1β, IL-6, and MCP-1 and increases in IL-10 production. Combination of oleic acid and keracyanin potentiate the results with a decrease in TLR4, IκKα, IκBα, NFκB1, MCP-1, TNF-α, IL-6, and IL-1β mRNA.
Delphinidin	2020	Imangali N. et al. [87]	In vivo—Wild-type and transgenic medaka (Oryzias latipes) osteoporosis model.	Delfinidin treatment dose-dependently inhibits RANKL-induced differentiation of macrophages into osteoclasts, decrease bone resorption.
Quercetin	2019	Dicarlo M. et al. [88]	In vivo—Mice.	Decrease in TNF-α expression in intestinal macrophages. Upregulation of the serine protease inhibitors in macrophages.
3-methyl-4′-glucuronate-resveratrol	2020	Peñalver P. et al. [89]	In vitro—LPS-activated RAW 264.7 macrophages cell-line.	Decrease in LPS-mediated activation of macrophages via inhibition of IL-6 and NO production. Downregulation of TNF-α gene expression.
23-hydroxy ursolic acid	2020	Ahn, Y. et al. [90]	In vivo—Female C57BL/6J mice. In vitro—bone marrow-derived macrophages (BMDM).	Decrease the activity of mitogen-activated protein kinase phosphatase-1 on 48% in BMDM from feed on high-calorie diet (nutrient-stressed monocytes) mice. Increase in conversion of macrophages into a transcriptionally hyperactive phenotype with higher anti-inflammation activity, including 10-fold decrease in IL-10 expression, increased GLUT 1 expression, normalized GLUT 3 and 4 genes expression.
Celastrol	2017	Luo D. et al. [91]	In vivo—Diet-induced obese C57BL/6N. In vitro—Murine RAW264.7 macrophages.	Reduce the levels of M1 macrophages IL-6, IL-1ß, TNF-α, iNOS production mRNA in vitro and in vivo by concentration-dependent suppression of LPS-induced activation of MAPK; time-dependent decrease in LPS-induced NFκB p65 subunit nuclear translocation and Nrf2-related induction of HO-1 expression.
Sulforaphane (SFR)	2022	Sun Y. et al. [39]	In vivo—Male C57BL/6Jnifdc mice (DSS colitis model). In vitro—BMDM from mice LPS and IFN-γ activated.	In vivo: Dose-dependent decrease in monocytes mucosa infiltration. Dose-dependent reduction in TNF-α, IL-1b, IL-6 levels and iNOS gene expression (M1) and increase in IL-10 levels and ARG-1, CD163, IL-10 and PTX-3 gene expression (M2). Decrease in F4/80+CD68+ (activated in colitis model) and boost of F4/80+CD206+. In vitro: Suppression of IL-1b and iNOS genes expression. Normalization of IL-10, PTX3 genes expression. Decrease in IL-1b, TNF-α, IL-6 production and increase in IL-10 levels by 2.5.
	2019	Jensen K. et al. [92]	In vitro—TBNC cell-line exposed to tumor-associated macrophages secretion.	SFR exposition significantly reduces tumor-associated macrophage proliferation.
Deoxyschizandrin and schizadrin from Schisandra chinensis berries	2018	Lee K. et al. [93]	In vitro—Human monocyte cell-line THP-1-stimulated and ovarian cancer cell-lines (to produce tumor associated M2 macrophages cell-line (CD163 and CD209)).	Significant suppression of CD163 and CD209 expression and suppression of mRNA expression with subsequent reduction in MMP-9, RANTES, VEGF production (tumor growth factors secreted by macrophages).

### 3.2. Dietary Fiber

In recent decades, the importance of sufficient dietary fiber has been investigated. Dietary fiber plays a crucial role in the microbiota functioning and maintaining biota species associated with low-grade inflammation reduction [94]. In the murine emphysema model, for instance, the high fiber reduces the macrophage infiltration into lungs tissue [59]. Dietary fiber inulin represents a form of liver protection from alcohol damage [60] because of a reliable effect on macrophage proliferation with promotion of M2 phenotype macrophages and suppression of M1 phenotype that is associated with SCFAs content [60]. SCFAs show multidirectional effects on macrophages. Thus, butyrate down-regulates the LPS-induced pro-inflammatory mediators’ levels [56] that are described in some studies as anti-inflammatory and lung-protective effects on emphysema [43] and influenza A [46,47] murine models. These effects can be partially explained by M2 macrophage phenotype proliferation stimulation [59,61,62].

### 3.3. Polyunsaturated (PUFAs) and Monounsaturated Fatty Acids

PUFAs and monounsaturated (MUFAs) fatty acids reliably decrease the TNF-α expression and LPS-induced inflammation proteins’ phosphorylation [35,63,64,65]. PUFAs express impressive anti-inflammatory properties. Monounsaturated oleic acid is able to potentiate effects of anthocyanins on pro- and anti-inflammatory cytokines mRNA expression and synthesis [35], whereas there is some controversy with regard to the effect of PUFAs on macrophage cholesterol metabolism depending on the types of studied macrophage cultures (human vs. murine) [66].

### 3.4. Vitamins

Vitamin D takes a significant role in immune response [67,68,95,96]. The deficiency of vitamin D is associated with the severity of the infection, e.g., COVID-19 [97]. On the contrary, vitamin D increases the resistance of macrophages to viruses and bacterial infections [67,68,70,71,72]. Vitamin D activates the localization of bacteria, reduces the pro-inflammatory LPS-stimulated M1 activation via MAPK-cascade inhibition and promotes the macrophages to M2 phenotype proliferation [67,68,69,70,71,72,73]. The last study also describes the results of specific binding of the vitamin D receptor on the surface of tumor-associated macrophages and proposes the possibility of antitumor immune response effects [98]. Overall, the potential effectiveness of vitamin D supplements as an immune-modulating agent is questionable and should be studied, but the importance of vitamin D deficiency elimination is undisputable.

Vitamin A also plays a crucial role in macrophage functioning. Macrophages participate in vitamin A activation from pro-vitamin A carotenoids [99]. Studies demonstrate the increase in anti-bacterial functioning [76] and decrease in pro-inflammatory macrophage reactions [74,75]. In cell culture of THP-1 monocyte/macrophages and in blood-derived mononuclear cells retinoic acid, the metabolite of vitamin A downregulated LPS-induced mRNA expression of pro-inflammatory cytokines IL-10, IL-12p40, TNF-α, IL-18, and TGF-β [81].

ABCA1, ATP-binding cassette transporter; ARG-1, arginase-1; ATRA, all-trans retinoic acids; BMDM, bone marrow-derived macrophages; CAMP, cathelicidin antimicrobial peptide; C3G, cyanidin-3-O-glucoside; CCL5, C-C motif chemokine ligand-5; COX-2, cyclooxygenase-2; CRIg, complement receptor immunoglobulin; DHA, docosahexaenoic acid; FAA, free fatty acid; GLUT, glucose transporter; IκBα, inhibitor NF-kB α; IKKα/β, inhibitory kappa B kinase α/β; IFN-γ, Interferon-γ; IL, interleukin; IRF-5, Interferon regulatory factor-5; iNOS, inducible nitric oxide synthase; JNK/SAPK p46, c-Jun N-terminal kinase/stress-activated protein kinase p46 subunit; 5-LOX, 5-lipoxygenase; LPS, lipopolysaccharide; MAPK, mitogen-activated protein kinase; MCP-1, monocyte chemoattractant protein-1; MMP, matrix metallopeptidase; miR-33, microRNA-33; NF-kB, nuclear factor kappa-B; NO, nitric oxide; NOS, nitric oxide synthase; NRF2, nuclear factor erythroid 2-related factor 2; P3G, peonidin-3-O-glucoside; PAI-1, plasminogen activator inhibitor-1; PGE2, prostaglandin E2; PGLYRT5, peptidoglycan recognition protein 5; PLA2, phospholipase A2; pNFκBp65, phosphorylated nuclear factor-kappaB p65 subunit; PPARγ, peroxisome proliferator-activated receptor γ; PTX-3, pentraxin-3; Pr3G, pelargonidin-3-O-glucoside; PUFAs, polyunsaturated fatty acids; RANKL, receptor activator of nuclear factor kappa-Β ligand; RANTES, regulated on activation, normal T cell expressed and secreted (CCL-5); RCA, red clover anthocyanins; SCFAs, Short-chain fatty acids; SOCS-1, suppressor of cytokine signaling-1; SREBP2, sterol regulatory element-binding protein; STD, standard-control diet; TGF-β, Transforming growth factor-β; TLR, Toll-like receptors; TNF-α, tumor necrosis factor-α; VEGF, vascular endothelial growth factor.

Previously known as vitamin B8, vitamin-like substance inositol hexaphosphate affects the macrophage proliferation and stimulates the anti-inflammatory 2A macrophages phenotypes [77].

### 3.5. Polyphenols

**Figure 1:**
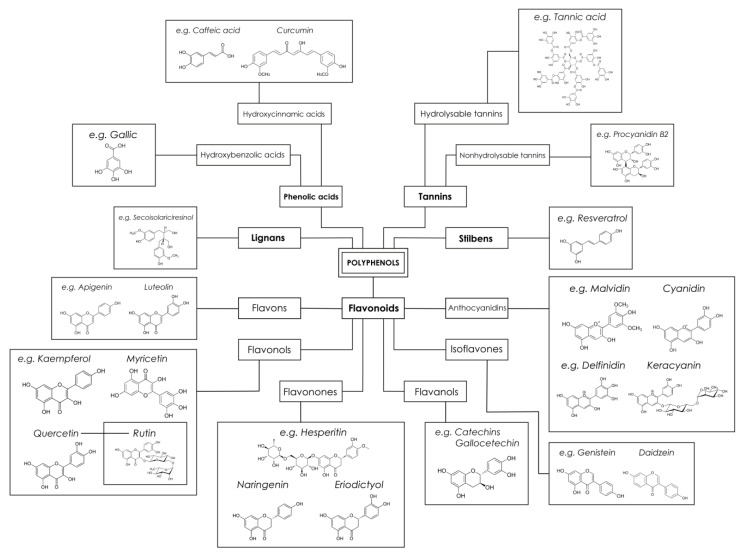
Polyphenols classification.

Polyphenols—a numerous group (Figure 1) of biologically active compounds with proven natural immune-modulatory properties [34] affecting the immune system in various pathways. Thus, olive oil polyphenols significantly reduce the expression of pro-inflammatory genes in the adipose tissue cell-model, including IL-1b, COX-2 and MCP-1 [100]. Another effect of polyphenols is a reduction in phosphorization of inflammation and oxidation-linked proteins, which underline the activation of pro-inflammatory cytokine secretion [82]. In another study, polyphenol-rich plant extract represents a potential preventive effect on macrophages adipose tissue infiltration [78]. In some research, the possible influence of polyphenols on cholesterol homeostasis and efflux in macrophages is predicted [101].

Curcumin is one of the most studied polyphenols from the phenolic acids subgroup. It promotes M2 phenotype proliferation and inhibits M1 proliferation [102] and foam cell formation [103], reduces LPS-stimulated expression of MIP-2, IL-1b, and 8 in macrophages [104].

Anthocyanins (a polyphenols subgroup) reduce the inflammation and ROS through enzymes binding [85] and blockage of pro-inflammatory signaling cascades [85,105]. The most investigated examples with anti-inflammatory properties are keracyanin [35] and delphinidin [52,87]. Additionally, several studies describe the indirect effect through the microbiota changes [106].

Quercetin, a widely described polyphenol from the flavanols subgroup, is able to reduce TNF-α genes expression and affect enzyme regulation [88]. Resveratrol, another polyphenol compound from stilbenes group, represents the same effect on TNF-α genes expression and is also capable of the inhibition of macrophage LPS-induced activation [89].

Overall, polyphenols are considered as one of the most important potential anti-inflammation nutraceuticals with evidence-based cell protection from an LPS-induced inflammation effect [79] (Table 2).

### 3.6. Non-Polyphenols Plant-Based Biologically Active Compounds

Non-polyphenols plant-based biologically active compounds with a reliable effect on macrophage functioning are pentacyclic triterpenoids hydroxyl ursolic acid and celastrol (an extract from roots, peels of fruits, berries, and herbs). The impact on macrophage enzymes’ activity and proliferation phenotyping of them causes the activation of anti-inflammation macrophages [90,91].

Sulforaphane (from cruciferous vegetables) is another phytochemical capable of reducing the pro-inflammation and tumor-associated macrophage proliferation, including the affection of cytokines genes expression [39,92]. A similar anti-tumor effect on tumor-associated macrophages is described for another plant-based biologically active substance—schizandrin [93].

## 4. Conclusions

This review was aimed to describe current studies presenting the role of diet in macrophage proliferation, differentiation, and functioning. According to the published data, diet patterns on the whole and some nutrients, e.g., MTD, dietary fiber and polyphenols, demonstrate a reliable influence on macrophages through numerous pathways. Further clinical research is demanded to develop and assess new diet recommendations, including diet prescription and scientifically proven supplementation designed for the correct functioning of macrophages and their introduction into real clinical practice—the last step from the bench to the bedside.

## Figures and Tables

**Table 1:** Recent studies which investigated the association of diet patterns with macrophage functioning.

Diet-Pattern	Year	Authors	Population	Brief Results
High-fat diet (HFD)	2020	Liu Y. et al. [16]	Mice model	Adipose tissue infiltration by M1-like macrophages (F4/80+CD11b+).
	2020	Liu T. et al. [17]	- 30 colorectal cancer patients, HFD vs. normal diet - Apc min/+ mice randomized to HFD or control	- Activation of MCP-1/CCR2 axis in intestinal macrophages, higher expression of CD163 M2 in human on HFD. - HFD promote M2 proliferation and decrease apoptosis–protumor effect.
High-salt diet (HSD)	2021	Hu M. et al. [18]	Patients with stroke, mice stroke models, in vitro and in vivo	Downregulation of expression of the myeloid cells 2 triggering receptor (TREM2) in macrophages that results in decreased efferocytic capacity and, subsequently, in neural pro-inflammation status and excessive stroke outcomes.
	2015	Zhang W.C. et al. [19]	Human, mice	Increase in LPS-induced activation of pro-inflammatory macrophages (M1) and decrease in IL-4-induced anti-inflammatory macrophages (M2) in lungs.
Protein or amino acids restricted diet	2018	Orillion A. et al. [20]	AA-restricted cultured RP-B6-Myc and RENCA mice TAM cell-lines and BMDM from C57BL/6 mice (prostate and kidney cancer model) feed protein-restricted diet	Decrease in M2-phenotype proliferation (pro-tumor type) in TAM and BMDM. In vitro M2 macrophages cultured with AA restriction lose their reducing effect on granzyme B expression and CD8+ T-cell. Decrease in secretion of the pro-tumor chemokynes—IL1ra, IL6, IL23, CXCL1, CCL5, and CCL17. Decrease in M2 macrophage capacity to phosphorylate STAT3 and increase in the phosphorylation of STAT1—M1 (tumoricidal) support. In vivo—promotion of effect of specific treatment or vaccine on tumor growth.
High-protein diet	2019	Kostovcikova K. et al. [21]	Immuno-competent BALB/c and immuno-deficient RAG2 knock-out mice on high animal protein diet vs. high plant protein diet	Higher expression of TGF-β, TNF-α and IL-1β in intestinal macrophages in animal protein group.
MTD	2021	Augimeri G. et al. [22]	Human, adolescence (N 77), human THP-1 monocytic cell-line (LPS-induced)	In vitro—reduction in IL-6 and TNF-α secretion in LPS-activated macrophages treated with serum from adolescence with high adherence to MTD.
Nordic diet	2019	Myhrstad M.C.W. et al. [23]	Randomized intervention study 18/24 week 68 participants with metabolic syndrome	Downregulation of 42 transcription gene factors (inc. NFR1, NFR2, NF-kB) of peripheral blood mononuclear cells on Nordic diet.
	2019	Ulven S.M. et al. [24]	SYSDIET sub-study 88 women 18/24 week	Downregulation of 48 transcription gene factors (inc. TNF and TNFRSF1A) and up-regulation of RELA proto-oncogene of fasting peripheral blood mononuclear cells on Nordic diet.
Dietary Approach to Stop Hypertension (DASH diet)	2021	Tahery A. et al. [25]	A cross-sectional study, 305 obese and overweight women, 18–48 y.o. DASH diet score assessment	No association between DASH diet score and MCP1 (monocyte chemoattractant protein 1) levels.

AA, amino acids; BMDM, bone marrow-derived macrophages; CCL, C-C motif chemokine ligand; CXCL1, C-X-C motif chemokine ligand 1; DASH, Dietary Approach to Stop Hypertension; IL, interleukin; LPS, lipopolysaccharide; MCP-1, monocyte chemoattractant protein-1; MTD, Mediterranean-type diet; NF-kB, nuclear factor kappa-B; TAM, tumor-associated macrophages; TGF-β, transforming growth factor-β; TNF-α, tumor necrosis factor-α; TNFRSF1A, TNF receptor superfamily member 1A; TREM2, triggering receptor expressed on myeloid cells 2; STAT, signal transducer and activator of transcription proteins.
